# Dietary Copper/Zinc Ratio and Type 2 Diabetes Risk in Women: The E3N Cohort Study

**DOI:** 10.3390/nu13082502

**Published:** 2021-07-22

**Authors:** Nasser Laouali, Conor-James MacDonald, Sanam Shah, Douae El Fatouhi, Francesca Romana Mancini, Guy Fagherazzi, Marie-Christine Boutron-Ruault

**Affiliations:** 1 Center for Research in Epidemiology and Population Health (CESP), Institut Gustave Roussy, U1018 Inserm, CEDEX, 94800 Villejuif, France; nasser.laouali@inserm.fr (N.L.); conor.macdonald@gustaveroussy.fr (C.-J.M.); Sanam.SHAH@gustaveroussy.fr (S.S.); Douae.EL-FATOUHI@gustaveroussy.fr (D.E.F.); Francesca.MANCINI@gustaveroussy.fr (F.R.M.); 2 Department of Biostatistics and Epidemiology, School of Public Health and Health Sciences, University of Massachusetts at Amherst, Amherst, MA 01002, USA; 3 Deep Digital Phenotyping Research Unit, Department of Population Health, Luxembourg Institute of Health (LIH), L-1445 Strassen, Luxembourg; guy.fagherazzi@gmail.com

**Keywords:** diet, copper, zinc, women, Type 2 diabetes, obesity, observational study, epidemiology

## Abstract

The serum copper (Cu) to zinc (Zn) ratio could be an important determinant of Type 2 diabetes (T2D) risk, but prospective epidemiological data are scarce. We aimed to investigate the association between T2D incidence and the dietary Cu/Zn ratio. A total of 70,991 women from the E3N cohort study were followed for 20 years. The intakes of copper and zinc were estimated at baseline using a validated food frequency questionnaire. We identified and validated 3292 incident T2D cases. Spline analysis showed that a Cu/Zn ratio < 0.55 was associated with a lower risk of T2D. Subgroup analyses comparing women in the highest versus the lowest quintile of Cu/Zn ratio showed the same pattern of association for obese women and those with zinc intake ≥8 mg/day. However, for women with zinc intake <8 mg/day, higher Cu/Zn ratio appeared to be associated with higher T2D risk. Our findings suggest that a lower dietary Cu/Zn ratio is associated with a lower T2D risk, especially among obese women and women with zinc intake >8 mg/day. Further studies are warranted to validate our results.

## 1. Introduction

Type 2 diabetes (T2D), a chronic disease resulting from complex gene–environment interactions, is becoming a major global health threat. A considerable body of evidence suggests that oxidative stress plays a key role in the development of T2D [1,2]. Moreover, trace elements such as copper (Cu) and zinc (Zn), essential minerals involved in many enzymatic processes and oxidant/antioxidant balance [3], have been linked to the pathogenesis of T2D [4]. The role of zinc in the pathophysiology of T2D has been widely reported in recent decades [5,6]; however, few studies have prospectively examined the association between dietary zinc and the risk of T2D, and have reported negative [7,8,9], positive [10], or null [11] results. By contrast, evidence for copper intake is limited; a prospective study reported a positive association with potential effect modification by age, smoking status, and family history of diabetes [9]. Taken together, these findings suggest an antagonistic relationship between copper and zinc, and the risk of diabetes; thus, studying suboptimal intakes of these trace elements may provide useful information. Therefore, we aimed to investigate the association between the dietary Cu/Zn ratio and T2D incidence in women, with potential effect modification by other factors.

## 2. Materials and Methods

### 2.1. Study Population and Follow-Up

The protocol of the E3N (*Etude Epidémiologique auprès de femmes de l’Education Nationale*) cohort, a French prospective cohort study, is available elsewhere [12,13]. Briefly, 98,995 women were selected from the French national health insurance plan for teachers and coworkers, the *Mutuelle Générale de l’Education Nationale*. Women were enrolled in the cohort through a self-administered questionnaire, and were biannually followed for health conditions, lifestyle, diet, treatments, and mental health status, etc. Furthermore, for each cohort member, the health insurance plan provided data that included all outpatient reimbursements for health expenditures since 1 January 2004; data included brand names, doses, and dates of drug reimbursements. The average response rate to a follow-up questionnaire was 83%, with a total loss to follow-up since 1990 was below 3%.

Eligible participants (*n* = 74,522) were those who completed the dietary questionnaire sent in 1993. We then excluded all prevalent T2D cases (*n* = 824), women with extreme energy intakes (i.e., below the 1st and above the 99th percentiles of the energy intake over energy requirement distribution in the population) (*n* = 1491), and women who did not complete any follow-up questionnaire after the dietary questionnaire (*n* = 1216). The final study population included 70,991 women. Follow-up started in 1993 (baseline for the present study) and ended in 2014 (latest date of T2D case validation in the E3N cohort).

The study was approved by the French National Commission for Data Protection and Privacy (ClinicalTrials.gov identifier: NCT03285230). All participants gave their written informed consent.

### 2.2. Copper and Zinc Intakes

Food intake was assessed using a validated 208-item semi-quantitative food frequency questionnaire sent in 1993 [14]. All food intakes were converted into intakes of energy and nutrients using a food composition database adapted from that of the French Information Center on Food Quality [15]. Energy-adjusted copper and zinc intakes were computed using the residual method as proposed by Willett et al. [16].

### 2.3. Ascertainment of Type 2 Diabetes

The procedure has been described in detail elsewhere [17,18]. Briefly, a diabetes-specific questionnaire was sent to all potential cases identified through follow-up questionnaires. Cases were defined as having at least one of the following: (1) fasting plasma glucose ≥7.0 mmol/L or random glucose ≥11.1 mmol/L at diagnosis; (2) use of a glucose-lowering medication; or (3) the most recent values of fasting glucose or HbA1c concentrations ≥7.0 mmol/L or ≥7%, respectively, in the diabetes-specific questionnaire. After 2004, case identification was based on the drug reimbursement insurance database. All women with at least two reimbursements for any glucose-lowering medication within a 1-year period were considered to be validated T2D cases, with the date of diagnosis defined as the date of first reimbursement.

### 2.4. Covariates

Covariates considered in this study were assessed at baseline (1993 questionnaire), or when not available, at the closest questionnaire. Body mass index (BMI), calculated by dividing weight in kilograms by height in squared meters, was considered continuously in all models, and in three categories for stratified analyses (<20 kg/m^2^, 20–24 kg/m^2^, and ≥25 kg/m^2^). The level of recreational physical activity (MET-h/week) was considered as a continuous variable. We considered three categories for smoking status (never, former, and current), educational level (undergraduate or less, graduate, and postgraduate or more), personal history of hypercholesterolemia (yes, no, and unknown), and two categories for family history of diabetes and personal history of hypertension (yes and no).

## 3. Statistical Analyses

The baseline characteristics of the participants, overall and according to quintiles of the Cu/Zn ratio, were expressed as means ± standard deviation (SD) for continuous variables, or numbers (percentages) for categorical variables.

We used Cox proportional hazards regression models with age as the time-scale to estimate hazard ratios (HR) and 95% confidence intervals (95% CI) for T2D risk. Participants were followed up from age at baseline until age at diagnosis of T2D, age at death, age at last follow-up questionnaire, or age at the end of the follow-up period (2014), whichever occurred first. Schoenfeld residuals and log-minus-log plots were examined to confirm the proportional hazards assumption. No major violations were observed in any model.

The Cu/Zn ratio was modeled in two different ways. For the main analysis, we used multivariable-restricted cubic splines with three knots placed at the 10th, 50th, and 90th percentiles of the Cu/Zn ratio distribution to provide a graphical presentation [19]. Splines allowed us to test whether there was a significant departure from a linear association. Second, we categorized the Cu/Zn ratio into quintiles and considered the 1st quintile group as the reference category in the Cox models.

Three models are presented, as follows: the first model was adjusted for age (time-scale); the second model was further adjusted for known T2D risk factors and potential confounding factors, including physical activity, smoking status, level of education, BMI, family history of diabetes, personal history of hypertension, and hypercholesterolemia; and the third model was further adjusted for total energy intake. The selection of potential confounders was done a priori, based upon the known risk factors of T2D available in our dataset and the association with the Cu/Zn ratio.

The interactions between the Cu/Zn ratio and BMI, age, smoking status, and family history of diabetes on the risk of T2D were tested by including a multiplicative term between the two variables in the Cox model, as well as by stratification on these variables. We also performed a further model adjusted for the following: (1) the ‘prudent’ diet characterized by high consumption of fruit, vegetables, legumes, fish, and poultry; and (2) the dietary intake of copper and zinc.

Statistical analyses were performed using SAS 9.4 (SAS Institute, Inc., Cary, NC, USA).

## 4. Results

### 4.1. Baseline Characteristics

Over a mean (SD) follow-up of 18.81 (4.3) years, a total of 3292 (4.6%) validated incident T2D cases were identified.

Table 1 presents the baseline characteristics of the study subjects, overall and according to quintiles of the Cu/Zn ratio. The mean (SD) daily intakes of copper, zinc, and the Cu/Zn ratio were 2.90 (1.15) mg, 11.51 (2.10) mg, and 0.26 (0.10), respectively. The mean age at baseline was 53 years (SD = 6.7). Higher Cu/Zn ratio was associated with older age, higher BMI and physical activity, and higher frequency of hypercholesterolemia at baseline. In addition, women who consumed a more ‘prudent’ diet had a higher Cu/Zn ratio (Table 1).

### 4.2. The Cu/Zn ratio and Type 2 Diabetes Risk

**Figure 1:**
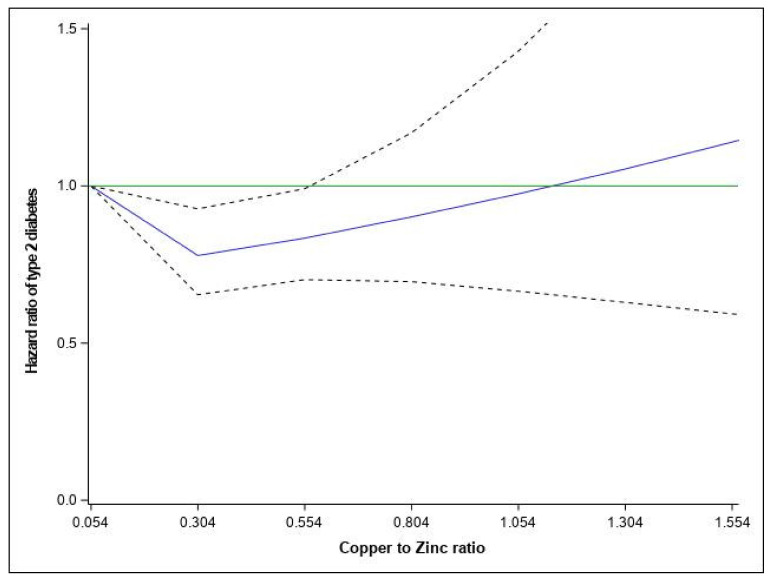
Relationship between dietary Cu/Zn ratio and hazard ratios of Type 2 diabetes fitted with restricted cubic splines (three knots placed at the 5th, 10th, and 50th percentiles). Risk estimates were adjusted for age, family history of diabetes, smoking status, physical activity, educational level, hypercholesterolemia, hypertension, body mass index, and total energy. Reference value for hazard ratios: minimum Cu/Zn ratio (0.054); the solid blue line represents the hazard ratio; the dashed lines the lower and upper 95% confidence interval. *p*_overall_ = 0.0194; *p*_NONLIN_ = 0.0173 and the solid green line represents the reference line of hazard ratio equal 1.

The association of the Cu/Zn ratio with T2D risk is presented in Table 2 and Figure 1. In the fully adjusted model, the Cu/Zn ratio was inversely associated with T2D risk from the 2nd (HR= 0.87 (95% CI: 0.78–0.97)) to the 3rd quintile (HR= 0.89 (95% CI: 0.79–0.99)) as compared to the 1st quintile group. Spline variables showed that there was departure from a linear association (*p* = 0.081), and a Cu/Zn ratio < 0.55 was associated with a lower risk of T2D (*p* = 0.0194) (Figure 1). When analyzed separately, the higher intakes of copper and zinc were associated with higher T2D risk (Supplementary Table S1).

There was an interaction between the Cu/Zn ratio and BMI on T2D risk (*p*_Interaction_ = 0.001), but not age (*p*_Interaction_ = 0.4798), smoking status (*p*_Interaction_ = 0.696), or family history of diabetes (*p*_Interaction_ = 0.1386) (data not tabulated). When stratified by BMI categories, the Cu/Zn ratio was inversely associated with T2D risk in obese women (for participants in the 2nd quintile compared to those in the 1st quintile, HR = 0.84 (95% CI: 0.72–0.97)), and positively associated with T2D in normal weight or overweight women (for participants in the 5th quintile compared to those in the 1st quintile, HR = 1.15 (95% CI: 0.93–1.43)) (Table 2).

In our study, 88% of women consumed more zinc than the recommended dietary allowance (RDA) (8 mg/day) and nearly all women (99%) consumed more copper than recommended (0.9 mg/day). When the analysis was stratified according to zinc RDA, we observed the same association between the Cu/Zn ratio and T2D in the group of women with zinc intake ≥8 mg/day as in the overall population. However, for women with zinc intake <8 mg/day higher Cu/Zn ratio appeared to be associated with higher T2D risk (Supplementary Table S2). Further adjustment for adherence to a ‘prudent’ diet and dietary intakes of zinc and copper did not change our results (data not shown).

## 5. Discussion

Our findings suggest a non-linear association between the baseline Cu/Zn ratio and T2D risk in women after adjusting for most known or potential risk factors or confounders. Women with low Cu/Zn ratio (<0.55) had lower T2D risk, especially for obese women and women with zinc intake >8 mg/day. For women with low zinc intake, higher Cu/Zn ratio was associated with higher T2D risk, though not significantly.

To the best of our knowledge, there is no prospective study about the association between dietary Cu/Zn ratio and T2D risk. The Cu/Zn ratio does not necessarily reflect the serum/plasma circulating levels; however, in line with our findings, previous studies reported that an imbalance between serum/plasma levels of copper and zinc were associated with altered glucose metabolism markers in obese patients with T2D [20]. Our results are also consistent with some clinical studies that reported a lower Cu/Zn ratio of serum/plasma concentrations in subjects without diabetes as compared to those with diabetes [21,22].

The mechanisms underlying the increased T2D risk associated with higher Cu/Zn ratio of serum/plasma concentrations are not well understood. Copper and zinc are needed for optimal activities of antioxidant enzymes; therefore, imbalance in the Cu/Zn ratio may influence the equilibrium in the antioxidant defense system and enhance the toxic effect of metal-dependent free radicals [21]. In addition, the imbalance of these elements might adversely affect the pancreatic islets and initiate and potentiate the pathogenic processes leading to T2D and its complications [23]. Excessive zinc intakes (150–450 mg/day) have been associated with chronic effects such as copper deficiency, altered iron function, reduced immune function, and reduced levels of high-density lipoproteins (HDL) [24]. In our study, 88% of women consumed more zinc than recommended (8 mg/day) [25] and nearly all women (99%) consumed more copper than recommended (0.9 mg/day); this could explain the observed high risk of T2D in this study population when the analysis was performed separately for each trace element. In the group of women with zinc intake ≥8 mg/day, the same U-shaped association between Cu/Zn ratio and T2D was observed as in the overall population, whereas for women with zinc intake <8 mg/day, a high Cu/Zn ratio appeared to be associated with higher T2D risk. However, the association was not statistically significant, likely due to the low power in the stratum of low zinc intake. Although our data did not concern the serum/plasma Cu/Zn ratio, we cannot exclude a similar mechanism for dietary intake ratio of Cu/Zn. Furthermore, the absorption of copper is strongly influenced by the amount of copper in the diet; the bioavailability ranges from 75% of dietary copper when the diet contains only 0.4 mg/day to 12% when the diet contains 7.5 mg/day [25]. With regard to zinc, the bioavailability depends on the source. The bioavailability from grains and plant foods is lower than that from animal foods, although many grain- and plant-based foods are good sources of zinc. It has been suggested that mechanisms leading to diabetes include high intakes of refined and energy-rich foods, which are presumed to be accompanied by suboptimal intakes of trace elements (5); in our study, women with high adherence to the ‘prudent’ dietary pattern had a higher Cu/Zn ratio, but adjustment for the prudent pattern did not change the results, which suggested that the potential effect of the Cu/Zn ratio is independent of the quality of diet.

A key limitation of our study is that our analyses are based on self-reported dietary intakes. In order to minimize the potential measurement error in the usual diet, we used validated tools [14], and excluded women with implausibly low- or high-energy diets. Because we only used dietary data from the 1993 questionnaire, we were not able to take into account changes in dietary habits over time. Another limitation of our study is the failure to consider zinc and copper supplementation, which can influence in part the variations of the circulating levels of these minerals. Lastly, the E3N cohort, like most cohort studies, is not representative of the French population. Few women were deficient in zinc and this is expected to be less likely in the French general population; therefore, extrapolating results to the general population must be done carefully.

## 6. Conclusions

In conclusion, our findings suggest a nonlinear association between the dietary Cu/Zn ratio and T2D risk. The Cu/Zn ratio <0.55 is associated with a lower T2D risk, especially among obese women and women with zinc intake >8 mg/day. The Cu/Zn ratio could be used as a biomarker to identify women susceptible to the risk of T2D if these results are confirmed. Further studies on dietary intakes and plasma level of Cu/Zn ratio are warranted to validate our results and to determine whether the associations are similar in men.

## Figures and Tables

**Table 1:** Baseline characteristics of the study population (E3N Cohort data, N = 70,991).

		Quintiles of Dietary Copper to Zinc Ratio				
	**Overall** **(N = 70,991)**	**Q1 (<0.18)** **(N = 14,198)**	**Q2 (0.18, 0.22)** **(N = 14,198)**	**Q3 (0.22, 0.27)** **(N = 14,198)**	**Q4 (0.27, 0.33)** **(N = 14,198)**	**Q5 (≥0.34)** **(N = 14,199)**
Type 2 diabetes diagnosis at the end of follow-up (%)	3292 (4.64)	672 (4.73)	634 (4.47)	619 (4.36)	668 (4.70)	699 (4.92)
Age at baseline (years)	52.88 (6.67)	53.24 (6.85)	53.01 (6.74)	52.86 (6.70)	52.76 (6.57)	52.53 (6.46)
Educational level (%)						
Undergraduate or less	7879 (11.09)	1482 (10.44)	1502 (10.58)	1520 (10.71)	1636 (11.52)	1739 (12.25)
Graduate	37,707 (53.12)	7473 (52.63)	7472 (52.63)	7586 (53.43)	7545 (53.14)	7631 (53.74)
Postgraduate or more	25,405 (35.79)	5243 (36.93)	5224 (36.79)	5092 (35.86)	5017 (35.34)	4829 (34.01)
Physical activity (MET-h/week)	49.37 (50.48)	46.72 (45.38)	48.50 (46.04)	49.40 (47.84)	51.10 (59.06)	51.11 (52.66)
Smoking status (%)						
Current	9581 (13.50)	1209 (8.52)	1536 (10.82)	1875 (13.21)	2072 (14.59)	2889 (20.35)
Former	23,257 (32.76)	4229 (29.79)	4546 (32.02)	4782 (33.68)	4927 (34.70)	4773 (33.62)
Never	38,153 (53.74)	8760 (61.69)	8116 (57.16)	7541 (53.11)	7199 (50.71)	6537 (46.03)
BMI (kg/m^2^)	22.89 (3.18)	22.60 (3.10)	22.78 (3.12)	22.87 (3.15)	23.04 (3.20)	23.14 (3.27)
BMI categories (%)						
<20 kg/m^2^	10,256 (14.45)	2390 (16.83)	2105 (14.83)	2082 (14.66)	1830 (12.89)	1849 (13.02)
20 to 24 kg/m^2^	46,872 (66.03)	9415 (66.31)	9515 (67.02)	9343 (65.81)	9427 (66.40)	9172 (64.60)
≥25 kg/m^2^	13,863 (19.52)	2393 (16.86)	2578 (18.15)	2773 (19.53)	2941 (20.71)	3178 (22.38)
Hypertension (%)	36,590 (51.54)	7179 (50.56)	7341 (51.70)	7352 (51.78)	7364 (51.87)	7354 (51.79)
Hypercholesterolemia (%)						
Yes	5030 (7.09)	1064 (7.49)	1024 (7.21)	1016 (7.16)	998 (7.02)	928 (6.54)
Unknown	7337 (10.34)	1410 (9.93)	1354 (9.54)	1428 (10.05)	1466 (10.33)	1679 (11.82)
Family history of diabetes (%)	7882 (11.10)	1495 (10.53)	1569 (11.05)	1580 (11.13)	1617 (11.39)	1621 (11.42)
Prudent dietary pattern	−0.01 (0.97)	−0.09 (0.92)	−0.01 (0.95)	−0.00 (0.97)	0.03 (0.98)	0.04 (1.05)
Copper (mg/day)	2.90 (1.26)	1.70 (0.45)	2.29 (0.53)	2.77 (0.65)	3.33 (0.82)	4.40 (1.45)
Zinc (mg/day)	11.51 (3.30)	11.86 (3.43)	11.79 (3.24)	11.64 (3.18)	11.44 (3.16)	10.80 (3.35)
Copper to zinc ratio	0.26 (0.10)	0.15 (0.02)	0.20 (0.02)	0.24 (0.02)	0.29 (0.02)	0.42 (0.09)

Data are presented as N (percent) for categorical variables and mean (standard deviation) for continuous variables.

**Table 2:** Risk of Type 2 diabetes according to quintile groups of dietary Cu/Zn ratio (E3N Cohort data, N = 70,991).

			M1	M2	M3
	Number (%) Non-Cases	Number (%) Cases	HR [95% CI]	HR [95% CI]	HR [95% CI]
Overall	N = 67,699	N = 3292			
Q1 (<0.18)	13,526 (19.98)	672 (20.41)	Reference	Reference	Reference
Q2 (0.18, 0.22)	13,564 (20.04)	634 (19.26)	0.95 [0.85; 1.06]	0.86 [0.77; 0.95]	0.87 [0.78; 0.97]
Q3 (0.22, 0.27)	13,579 (20.06)	619 (18.80)	0.94 [0.84; 1.04]	0.85 [0.76; 0.95]	0.89 [0.79; 0.99]
Q4 (0.27, 0.33)	13,530 (19.99)	668 (20.29)	1.02 [0.91; 1.13]	0.88 [0.79; 0.98]	0.94 [0.84; 1.06]
Q5 (≥0.34)	13,500 (19.94)	699 (21.23)	1.09 [0.98; 1.21]	0.91 [0.82; 1.01]	1.06 [0.93; 1.22]
BMI < 20 kg/m^2^	N = 10,162	N = 94			
Q1 (<0.18)	2320 (22.83)	23 (24.47)	Reference	Reference	Reference
Q2 (0.18, 0.22)	2090 (20.57)	17 (18.09)	0.82 [0.44; 1.54]	0.79 [0.42; 1.47]	0.88 [0.47; 1.66]
Q3 (0.22, 0.27)	2024 (19.92)	15 (15.96)	0.76 [0.40; 1.46]	0.70 [0.36; 1.34]	0.87 [0.44; 1.72]
Q4 (0.27, 0.33)	1848 (18.19)	17 (18.09)	0.96 [0.51; 1.80]	0.92 [0.49; 1.73]	1.29 [0.64; 2.58]
Q5 (≥0.34)	1880 (18.50)	22 (23.40)	1.21 [0.67; 2.17]	1.04 [0.57; 1.90]	1.83 [0.85; 3.96]
BMI 20–24 kg/m^2^	N = 45,488	N = 1384			
Q1 (<0.18)	9125 (20.06)	290 (20.95)	Reference	Reference	Reference
Q2 (0.18, 0.22)	9206 (20.24)	277 (20.01)	0.95 [0.81; 1.13]	0.94 [0.80; 1.11]	0.96 [0.82; 1.14]
Q3 (0.22, 0.27)	9119 (20.05)	275 (19.87)	0.96 [0.81; 1.13]	0.93 [0.79; 1.10]	0.97 [0.82; 1.16]
Q4 (0.27, 0.33)	9156 (20.13)	258 (18.64)	0.90 [0.76; 1.07]	0.88 [0.74; 1.04]	0.95 [0.79; 1.15]
Q5 (≥0.34)	8882 (19.53)	284 (20.52)	1.05 [0.89; 1.24]	0.99 [0.84; 1.17]	1.15 [0.93; 1.43]
BMI ≥ 25 kg/m^2^	N = 12,049	N = 1814			
Q1 (<0.18)	2073 (17.20)	367 (20.23)	Reference	Reference	Reference
Q2 (0.18, 0.22)	2284 (18.96)	324 (17.86)	0.82 [0.70; 0.95]	0.82 [0.70; 0.95]	0.84 [0.72; 0.97]
Q3 (0.22, 0.27)	2403 (19.94)	363 (20.01)	0.87 [0.75; 1.00]	0.86 [0.74; 0.99]	0.90 [0.78; 1.05]
Q4 (0.27, 0.33)	2535 (21.04)	384 (21.17)	0.87 [0.75; 1.00]	0.84 [0.73; 0.97]	0.92 [0.78; 1.08]
Q5 (≥0.34)	2754 (22.86)	376 (20.73)	0.81 [0.70; 0.93]	0.78 [0.67; 0.90]	0.91 [0.76; 1.10]

M1: Adjusted for age (as the time-scale); M2: M1+ family history of diabetes, smoking status, physical activity, educational level, hypercholesterolemia, hypertension, and body mass index (except for the stratified analysis); M3: M2+ total energy.

## Data Availability

The datasets generated during and/or analyzed for the current study are available from the corresponding author on reasonable request.

## Supplementary Materials

The following are available online at https://www.mdpi.com/article/10.3390/nu13082502/s1, Table S1: Risk of Type 2 diabetes according to quintile groups of dietary Cu and Zn (E3N Cohort data, N=70,991), Table S2: Risk of Type 2 diabetes according to quintile groups of dietary Cu -Zn ratio (E3N Cohort data, N=70,991). Please check Tables S1 and S2 in the Supplementary File.
