# Supplementary material for: Dietary Copper/Zinc Ratio and Type 2 Diabetes Risk in Women: The E3N Cohort Study
Source: Nutrients. 2021 Jul 22;13(8):2502. doi: 10.3390/nu13082502 (PMC8400926; doi:10.3390/nu13082502)
Supplement: Supplementary file 1 [file nutrients-13-02502-s001.zip › nutrients-1250109-supplementary.pdf]

Table S1. Risk of Type 2 diabetes according to quintile groups of dietary Cu and Zn (E3N Cohort data, N=70,991).

	**Number (%) non-cases**	Number (%) cases	M1 HR [95% CI]	M2 HR [95% CI]	M3 HR [95% CI]
**Copper**	N=67699	N=3292			
Q1 (< 1.99)	13595 (20.08)	604 (18.35)	Reference	Reference	Reference
Q2 (1.99,2.45)	13581 (20.06)	617 (18.74)	1.01 [0.90; 1.13]	0.98 [0.87; 1.09]	1.03 [0.92; 1.16]
Q3 (2.45,2.94)	13600 (20.09)	598 (18.17)	0.99 [0.88; 1.10]	0.86 [0.77; 0.96]	0.95 [0.84; 1.07]
Q4 (2.94,3.67)	13523 (19.98)	675 (20.50)	1.14 [1.02; 1.27]	0.96 [0.86; 1.07]	1.10 [0.98; 1.24]
Q5 (≥ 3.67)	13400 (19.79)	798 (24.24)	1.40 [1.26; 1.56]	1.01 [0.91; 1.13]	1.25 [1.10; 1.43]
**Zinc**	N=67699	N=3292			
Q1 (< 9.85)	13715 (20.26)	483 (14.67)	Reference	Reference	Reference
Q2 (9.85,10.81)	13682 (20.21)	516 (15.67)	1.07 [0.94; 1.21]	0.99 [0.88; 1.12]	1.01 [0.89; 1.14]
Q3 (10.81,11.74)	13499 (19.94)	700 (21.26)	1.47 [1.31; 1.65]	1.20 [1.07; 1.35]	1.23 [1.09; 1.38]
Q4 (11.74,13.00)	13464 (19.89)	734 (22.30)	1.55 [1.38; 1.74]	1.24 [1.10; 1.39]	1.27 [1.13; 1.42]
Q5 (≥ 13.00)	13339 (19.70)	859 (26.09)	1.86 [1.66; 2.08]	1.34 [1.19; 1.50]	1.35 [1.21; 1.52]

M1: Adjusted for age (as the time-scale)

M2: M1+ family history of diabetes, smoking status, physical activity, educational level, hypercholesterolemia, hypertension , and body mass index

M3: M2+ total energy

Table S2. Risk of Type 2 diabetes according to quintile groups of dietary Cu -Zn ratio (E3N Cohort data, N=70,991).

	**Number (%) non-cases**	Number (%) cases	M1 HR [95% CI]	M2 HR [95% CI]	M3 HR [95% CI]
**Zn ≤ 8 mg/day**	N=8560	N=330			
Q1 (< 0.18)	1369 (15.99)	56 (16.97)	Reference	Reference	Reference
Q2 (0.18,0.22)	1292 (15.09)	59 (17.88)	1.10 [0.76; 1.59]	0.95 [0.65; 1.37]	1.05 [0.72; 1.53]
Q3 (0.22,0.27)	1435 (16.76)	47 (14.24)	0.80 [0.54; 1.18]	0.76 [0.51; 1.12]	0.89 [0.60; 1.34]
Q4 (0.27,0.33)	1679 (19.61)	62 (18.79)	0.93 [0.65; 1.34]	0.85 [0.59; 1.22]	1.07 [0.71; 1.60]
Q5 (≥ 0.34)	2785 (32.54)	106 (32.12)	1.00 [0.72; 1.38]	0.86 [0.62; 1.20]	1.27 [0.81; 1.99]
**Zn > 8 mg/day**	N=59139	N=2962			
Q1 (< 0.18)	12157 (20.56)	616 (20.80)	Reference	Reference	Reference
Q2 (0.18,0.22)	12272 (20.75)	575 (19.41)	0.94 [0.83; 1.05]	0.85 [0.76; 0.95]	0.86 [0.77; 0.97]
Q3 (0.22,0.27)	12144 (20.53)	572 (19.31)	0.95 [0.85; 1.06]	0.86 [0.77; 0.96]	0.89 [0.79; 1.01]
Q4 (0.27,0.33)	11851 (20.04)	606 (20.46)	1.03 [0.92; 1.15]	0.88 [0.79; 0.99]	0.94 [0.83; 1.07]
Q5 (≥ 0.34)	10715 (18.12)	593 (20.02)	1.15 [1.02; 1.28]	0.93 [0.83; 1.04]	1.07 [0.93; 1.24]

M1: Adjusted for age (as the time-scale)

M2: M1+ family history of diabetes, smoking status, physical activity, educational level, hypercholesterolemia, hypertension , and body mass index

M3: M2+ total energy
